# Retraction of Sorting protein VPS33B regulates exosomal autocrine signaling to mediate hematopoiesis and leukemogenesis

**DOI:** 10.1172/JCI197214

**Published:** 2025-08-15

**Authors:** Hao Gu, Chiqi Chen, Xiaoxin Hao, Conghui Wang, Xiaocui Zhang, Zhen Li, Hongfang Shao, Hongxiang Zeng, Zhuo Yu, Li Xie, Fangzhen Xia, Feifei Zhang, Xiaoye Liu, Yaping Zhang, Haishan Jiang, Jun Zhu, Jiangbo Wan, Chun Wang, Wei Weng, Jingjing Xie, Minfang Tao, Cheng Cheng Zhang, Junling Liu, Guo-Qiang Chen, Junke Zheng

Original citation: *J Clin Invest*. 2016;126(12):4537–4553. https://doi.org/10.1172/JCI87105

Citation for this retraction: *J Clin Invest*. 2025;135(16):e197214. https://doi.org/10.1172/JCI197214

The Editors recently became aware of errors in Figure 1F, Figure 2D, Supplemental Figure 1H, and Supplemental Figure 7J. Although the authors have indicated that original source data support the findings in these figure panels, the Editors are retracting the article due to the number of errors made.

Hao Gu, Chiqi Chen, Xiaoxin Hao, Xiaocui Zhang, Zhen Li, Hongfang Shao, Hongxiang Zeng, Zhuo Yu, Li Xie, Fangzhen Xia, Feifei Zhang, Yaping Zhang, Haishan Jiang, Jun Zhu, Wei Weng, Minfang Tao, Cheng Cheng Zhang, Junling Liu, Guo-Qiang Chen, and Junke Zheng dissent from retraction.

Conghui Wang, Xiaoye Liu, Jiangbo Wan, Chun Wang, and Jingjing Xie could not be reached.

## Version 1 08/15/2025

Electronic publication

